# Imagining alternative futures with augmentative and alternative communication: a manifesto

**DOI:** 10.1136/medhum-2024-013022

**Published:** 2024-10-31

**Authors:** Darryl Sellwood, Lateef McLeod, Kevin Williams, Katie Brown, Graham Pullin

**Affiliations:** 1 Flinders University, Adelaide, South Australia, Australia; 2 California Institute of Integral Studies, Oakland, California, USA; 3 Independent Researcher, Charlotte, North Carolina, USA; 4 Duncan of Jordanstone College of Art and Design, University of Dundee, Dundee, Angus, UK

**Keywords:** Medical humanities, disability, Digital Technology

## Abstract

This manifesto seeks to challenge dominant narratives about the future of augmentative and alternative communication (AAC). Current predictions are mainly driven by technological developments—technologies usually being developed for different markets—and are often based on ableist assumptions. In online conversations and a discussion panel at the 2023 International Society for Augmentative and Alternative Communication conference, we explored alternative futures by adopting different starting positions. Our case is presented under five headings: questioning the dominance of predictions that artificial intelligence and brain-computer interfaces will define the future of AAC; resisting disability being framed medically, as a problem to be solved, yet acknowledging both the pleasures and pains of being disabled; declaring that people who use AAC—as cyborgs of necessity rather than choice—should have choice and ownership of our technologies; challenging notions of independence as the necessary end goal for disabled bodies and considering interdependence as a human right; imagining alternative futures in which all people who use AAC are accepted and embraced for our communication and self-expression. This manifesto is an invitation for further discussion, and we welcome responses. While our focus is AAC, and three of the authors use AAC, we believe that our stance could be relevant to other disability communities in turn. This paper is about who gets to imagine disability futures and whose voices are left out. It is about how uncritical these futures can be, often presuming values that disabled people, in all their diversity, may not share.

 We propose a new approach to imagining futures with augmentative and alternative communication (AAC). At a time when diversity is increasingly valued, we feel our future is nevertheless being determined by technology; technologies that are not as ‘neutral’ as supposed, but which embody values that can go unquestioned.

We would like futures to be framed not by new technologies but by principles of social inclusion —and for these futures to be conceived and owned by people who use AAC. (*: the language we have chosen to use, the coauthors’ own use of AAC and the process of writing this manifesto are described at the end of the document).

To *briefly* introduce what we mean by AAC, it encompasses many ways that people communicate who do not, or do not always, use direct speech. Speech-generating devices may be involved, as may vocalising, revoicing, gesturing and body language. These and more may be used in combination and in different contexts. People who use AAC may do so primarily or intermittently.

Our thinking is built on other manifestos about disability and/or technology, projected onto our lived experience. Although people who have used AAC for a long time have their preferred tools and methods, worked out by a process of discovery, the future of AAC feels even less directed by disabled people themselves than other areas of disability. Yet we believe that our particular stance could be relevant to other disability communities.

We present our case under the following statements:

1. We question the dominance of predictions that artificial intelligence (AI) and brain-computer interfaces (BCIs) will define the future of AAC.
2. We resist disability being framed medically, as a problem to be solved; yet we acknowledge both the pleasures and pains of being disabled.
3. We declare that people who use AAC—as cyborgs of necessity rather than choice—should have choice and ownership of our technologies.
4. We challenge notions of independence as the necessary end goal for disabled bodies and consider interdependence as a human right.
5. We imagine alternative futures in which all people who use AAC are accepted and embraced for our communication and self-expression.

## Questioning dominant technological predictions

We question the dominance of predictions that AI and BCIs will define the future of AAC.

One imagined future is firmly represented in the public domain: front page newspaper stories herald the advent of brain implants offering ‘speech at the speed of thought’ (The Guardian 2019). If approached with care these technologies offer real potential (Peters *et al* 2022). Another article about developments in implanted BCI is entitled ‘Tapping into the brain to help a paralysed man speak’ (New York Times 2021). In the way in which it describes this man as being helped, rather than also actively helping himself, we feel that the article reinforces a charity model of disability that considers disabled people as objects of pity and a burden on society (Berkeley Disability Lab 2024). In the article, when the algorithm successfully identified a sentence that the man intended, he appears pleased; for repetitive tasks, or when the algorithm fails, he is reported as appearing distracted or unmotivated. Successes are attributed to the technology, while shortcomings in reliability are ascribed to the person using the technology. This serves to downplay his labour in training the algorithm and device. ‘BCI Illiteracy’ is a recognised but sticky term in BCI research. ‘BCI illiteracy assumes a problematic framing: that the user possesses a state or quality of deficit that may be discovered through their interaction and poor performance on a BCI system’ (Thompson 2019). BCI media coverage neglects to acknowledge the enormous amount of disability labour that is required in mastering these technologies.

While implanted BCIs are not yet broadly available, clinical applications are fast approaching and there is a growing body of research about non-invasive worn BCI devices. As discussed in a recent systematic review of BCIs with AAC applications, the success of these devices varies (Peters *et al* 2022). The user interactions tend to be predominantly textual, which may limit participation in studies to those deemed literate. These devices offer huge promise for those living with conditions such as amyotrophic lateral sclerosis. However, the review warns researchers to ‘exercise caution in interpreting the results of studies in which participants without disabilities test technology intended for people with disabilities’ (Peters *et al* 2022).

BCIs currently dominate the conversation as being *the* future of AAC. Meanwhile, use of low-tech and no-tech approaches continues worldwide. Even when developed, the high cost of BCI will make it unavailable to many disabled people, just as eye-gaze technology remains unaffordable to many people who use AAC, highlighting the economic inequality around assistive technology.

AI is also heralded as being poised to revolutionise everything, although leading researchers have pointed out that its history in AAC is much longer and more gradual (Waller 2024). Predictive text in speech-generating devices can already alleviate manual typing demands and location-based communication suggestions can be helpful in quickly accessing common phrases. Others discussing the potential and implications of AI have raised precautions that biases within these technologies could compromise autonomy and individuality, for example, if machine learning models were consistently generating words or phrases that were culturally or geographically inappropriate (Sennott *et al* 2019). Who controls the vocabulary available through the technology? How does that shape what people are able to say? How do we tell if what is being communicated accurately reflects what was intended? Will individuals simply defer to the programmed algorithm or will they be able to genuinely express themselves as they desire?

We are concerned by the tendency to paint these technologies as neutral or positive. Shew (2023) coined the term ‘technoableism’ to describe the non-critical framing of technology as a ‘solution’ to the ‘problem’ of disability. Technoableism can be interpreted in the narrative disseminated by certain companies developing BCI, who position their technology as a potential ‘cure’ for disabilities including paralysis and deafness. Proposing alternative futures for AAC, we suggest these technological advancements are not cures or solutions, but rather assistive tools for multimodal communication.

## Resisting a medical framing of disability

We resist disability being framed medically, as a problem to be solved; yet we acknowledge both the pleasures and pains of being disabled.

The medical model of disability pathologises disabled bodyminds (Kafer 2013). We favour framing understanding through social (Shakespeare 2018) or biopsychosocial models of disability, where disability is understood not as an individual problem but as a contextually dependent state of being. A design model of disability is also illuminating, positioning disability not as a problem to be solved but as an inherently creative way of being in the world (Guffey and Williamson 2020).

A medical model is implied by framing complex communication needs as a condition to be fixed through surgical intervention—and ‘cosmetically invisible’ brain implants (Neuralink), to be hidden within a normalised bodymind (Kafer 2013). A top-down approach by technology developers means important decisions of what technology could do are not under the control of those who do or will use the technologies. Without rejecting its potential, we still ask whether brain to speech technology is the future that every augmented communicator wants. What about disabled people who do not need to be cured or fixed, because they do not see themselves as being broken?

Current news coverage of advancements in BCIs mirror the often simplistic marketing of cochlear implants (CIs) as cures for deafness. The social model of disability can be used to challenge the deficit stance of deafness portrayed by the medical model. Lane and Ladd note that shared sign language and politics creates a culture of Deafness and coin the term Deafhood as an alternative, positive framing of Deaf ontology, opposite to that of hearing loss as an ontology centred on deficit (Ladd and Lane 2013).

As neural prostheses, CIs could be seen as precursors to brain-to-speech interfaces. However, it is well documented that CIs are not simple solutions to deafness (Blume 2010; Campbell 2009; Mauldin 2016). Rather CI wearers are working *with* these technologies, learning to process sound perhaps for the first time via the brain, and training the brain to make sense of sound requires substantial labour on the part of the person being implanted.

Despite positive framings of deafness through Deafhood, the existence of CI and other BCI technologies means the act of refusing technological intervention can be viewed as a contentious choice by the medical field. Instead of being viewed as a cure, both BCIs and CIs should be viewed as potentially beneficial technologies that still have controversial challenges, like their invasiveness, that must be considered.

## Being cyborgs of necessity not choice

We declare that people who use AAC—as cyborgs of necessity rather than choice—should have choice and ownership of our technologies.

Jillian Weise’s essay ‘Common Cyborg’ reflects critically on cyborg status (Weise 2018), defining this as a disability identity earned through lived experience with body-extending or augmenting technologies. We consider that people who use AAC are similarly cyborgs, not through choice—unlike non-disabled transhumanists (whom Weise terms ‘tryborgs’)—but through societal pressures of not being understood any other way but by adopting technology to communicate. We argue that even though current AAC technology is outside the body, it is analogous to Weise’s own prosthetic leg. If a person uses AAC, the technology can become a part of their being. Correspondingly, if a person doesn't fully buy in to this assimilation, they won't use it effectively. Technology breakdowns and upgrades can be devastating. So now and in the future, people who use AAC can claim cyborg identity without needing to implant BCI devices.

Disabled people need to be the ultimate authority in what technology works for them and what does not. Medical professionals should not assume and decide what is best for users. Hartblay (2020) discusses how disabled people gain expertise through their lives on what works for them to navigate the world. Disabled people gain this knowledge through life experiences. These unique perspectives cannot be easily obtained by non-disabled people. Consequently, disabled people are best suited to know if certain technologies are right for them and fit with their lifestyles. Healthcare and assistive technology specialists should honour knowledge produced through lived experience as fact and trust disabled people to express what technologies are right for them. In AAC, these technologies currently include speech-generating devices, typically on screen-based tablets, sometimes augmented by inputs such as joysticks and eye-tracking. Yet, AAC also includes low-tech and no-tech methods such as communication boards, gestures, body language and revoicing via human communication assistants.

In several disability cultures, members of the community are positioned in places of authority in the assistive technology field, however in AAC this type of authority is lacking. Choice of device, voice and access method needs to be devolved and democratised. The AAC field has shifted in recent decades from dedicated devices towards the use of apps running on more mainstream products. While this is in some ways inclusive, there remain positive aspects of AAC devices that are recognisable as AAC.

While AAC technologies are often promoted as a miracle cure for speech disabilities, the substantial effort required to learn and use them effectively is often overlooked. Users frequently must abandon AAC devices they have mastered due to discontinued support, requiring them to investigate and adopt alternative solutions. Like any new technology, AAC requires learning and adaptation, but people who use AAC must also develop extra skills to ensure accessibility and usability. They often need to find creative solutions to new barriers introduced by the new technology just to maintain their previous level of participation in their environment. Therefore, disabled people should be the ultimate authority on which technologies are effective for them.

## Challenging independence and advocating interdependence

We challenge notions of independence as the necessary end goal for disabled bodies and consider interdependence as a human right.

While independent communication remains a key objective and a measure of success within the AAC field, is the notion of an ‘independent communicator’ fundamentally contradictory? Communication implies an interactive exchange between two or more parties and is, by definition, co-constructed between communicators. Otherwise, it is a soliloquy. Revoicing a person’s dysarthric speech is as much a part of AAC as is text-to-speech software. Why is this type of support for communication so often dismissed and people told to use their AAC devices? Communication breakdowns are evident when watching conversations play out between people. Whereas when these breakdowns involve AAC, faults in communication are often wrongly ascribed to the person using AAC rather than to the technology or their conversational partners.

In challenging these assumptions, we draw from and build on critical activism including the ‘10 Principles of Disability Justice’: intersectionality, leadership of those most impacted, anticapitalism, cross-movement organising, wholeness, sustainability, cross-disability solidarity, interdependence, collective access and collective liberation (Sins Invalid 2015). And the ‘Crip Technoscience Manifesto’ which lays out four commitments: centring the work of disabled people as knowers and makers, committing to access as friction, committing to interdependence as political technology, and committing to disability justice (Hamraie and Fritsch 2019).

So we challenge the notion of the ‘ideal worker’ as an able-bodied, white, independent entity. We argue that independence is an inherently capitalist and flawed goal for humanity writ large and inevitably unattainable. Independence is a myth, a state that non-disabled people tell themselves that they have achieved—while other people grow their food, make their clothes and build their houses. Ultimately, as the social model of disability infers, society was built originally for one version of the bodymind and we now need to take the total spectrum of human variation into consideration as we conceive new disabled futures of liberation.

We resist a future framed around barely veiled eugenic, ableist, white supremacist principles; technology positivist transhumanist narratives that frame the future as an imagined space of ‘no future’ for disabled bodyminds (Kafer 2013). We remind our readers that the future is disabled (Piepzna-Samarasinha 2022).

## Accepting and embracing communication with AAC

We imagine alternative futures in which all people who use AAC are accepted and embraced for our own communication and self-expression.

AAC applications still seem to be fixated on a sender-receiver model that prioritises spoken language above all else (Higginbotham *et al* 2016). We can find solidarity with the Deaf community in positing that spoken language need not be the only form of language that is legitimised in AAC. If people’s views on viable forms of communication stay rigid, then how can they fully accept AAC as legitimate communication, equal to ‘normative’ oral speech? One of the roles of disability studies is to question the assumptions of ‘normal’ communication. We ask for the diversity of technologies, and methods and styles of communication preferred by people who use AAC to be more fully embraced.

Many people with disabilities are excellent problem solvers—and have to be. But this expertise is so often overlooked or undervalued. Disabled people might be asked to sit on consumer panels or have a representative seat on the board (often voluntary or with some token payment). But we need more people with disabilities to be actively involved in designing and developing solutions to the real-world problems that affect us. We need more people who use AAC on the boards of companies that matter to them. We need funding opportunities and support within academia and industry not as participants or patients, but as researchers, developers and project leaders.

AAC as a cultural group is still young and we are finding our own voice that is separate and sometimes opposed to the now standard voices in the AAC community, which are predominantly well-meaning researchers and clinicians. As more people who use AAC take the lead on community initiatives and research projects, the AAC field is expanding beyond finding pathways to speak, to encompass economic, social and political impacts. We encourage all those who interact with people who use AAC to engage with these perspectives, that are also at the heart of disability studies.

To have a liberated disability justice future we not only need disabled people innovating and developing the science and technology that will help them have better lives in the future, but also disabled artists, writers, philosophers and social scientists that will hypothesise new ideas of how we see ourselves that will incorporate disabled ways of being and knowing. Using this disabled epistemology is one way we can direct the path to a more liberated world.

## Creating the manifesto and asking for a response

This manifesto challenges the prevailing narratives about the future of AAC, which are predominantly driven by technological advancements often rooted in ableist assumptions. Through discussions in the field of AAC, we have developed this dissenting position. We argue that the future of AAC should not be dictated by new technologies but by principles of social inclusion and equity, ensuring that augmented communicators have a say in shaping their own futures. While technological innovations like AI and BCIs receive significant attention, they often come with biases and assumptions that are counter to the lived experience of people who use AAC. For instance, the portrayal of technologies as solutions frequently overlooks the substantial effort required to take up and use these technologies. Similarly, the push for people who use AAC to have independence in their communication overlooks the essential human right of interdependence, crucial for authentic and meaningful communication.

Throughout this manifesto we have combined identity-first language around disability: ‘disabled people’, with person-first language around AAC: ‘person who uses AAC’. The former makes a connection with other disability activism; the latter draws on research into language around AAC (Zisk and Konyn 2022). We have been consistent for the sake of coherence, although we acknowledge different usages of these terms across disciplines and even different preferences within the field of AAC (Sellwood 2019).

Three of the authors use AAC, including vocalising, revoicing and speech-generating devices and applications, sometimes in combination, and provide leadership in the community. Two of the authors are design researchers bringing creative and collaborative methodologies to AAC research. We are all active in discussions of AAC around intersectional issues, relationships and other relevant topics. Together we have written this manifesto as a piece of collective self-expression in creating a vision for AAC culture.

The catalyst for the manifesto was a discussion panel held at the International Society for Augmentative and Alternative Communication conference in Cancun, Mexico in July 2023. The title was 'Imagining alternative futures with AAC: what interactions do we want with each other?' (Pullin *et al* 2023). The themes that emerged from this discussion were not only technology and interactions but also *ownership* of AAC and of its future as a field. This more political perspective suggested the format of a manifesto, a practice that we have employed in previous disability research. The process of coauthoring involved shared files, appropriately asynchronous to our geographical distance and also our different communication preferences. An online workshop and symposium provided opportunities for conversation and connections with scholarship about other disabilities and technologies.

Drawing on lived experience and existing frameworks from disability justice and crip technoscience, this manifesto calls for a shift from a top-down development approach, born from a medical model of disability, towards a more inclusive, socially aware and person-centred approach. It emphasises the importance of disabled people directing the development of AAC technologies, including decision-making at every level. Disruptive disability futures are needed, in which the diverse voices of people who use AAC are integral. This manifesto is an invitation for ongoing dialogue and action.

## Data Availability

There are no data in this work.
